# Resources and Methods for Engineering “Designer” Glycan-Binding Proteins

**DOI:** 10.3390/molecules26020380

**Published:** 2021-01-13

**Authors:** Ruben Warkentin, David H. Kwan

**Affiliations:** 1 Department of Biology, Centre for Applied Synthetic Biology, and Centre for Structural and Functional Genomics, Concordia University, 7141 Sherbrooke Street West, Montreal, QC H4B 1R6, Canada; ruben.warkentin@mail.concordia.ca; 2 PROTEO, Quebec Network for Research on Protein Function, Structure, and Engineering, Quebec City, QC G1V 0A6, Canada; 3 Department of Chemistry and Biochemistry, Concordia University, 7141 Sherbrooke Street West, Montreal, QC H4B 1R6, Canada

**Keywords:** glycobiology, glycan-binding protein, lectins, protein engineering, glycans, carbohydrates, directed evolution, glycan immobilization

## Abstract

This review provides information on available methods for engineering glycan-binding proteins (GBP). Glycans are involved in a variety of physiological functions and are found in all domains of life and viruses. Due to their wide range of functions, GBPs have been developed with diagnostic, therapeutic, and biotechnological applications. The development of GBPs has traditionally been hindered by a lack of available glycan targets and sensitive and selective protein scaffolds; however, recent advances in glycobiology have largely overcome these challenges. Here we provide information on how to approach the design of novel “designer” GBPs, starting from the protein scaffold to the mutagenesis methods, selection, and characterization of the GBPs.

## 1. Introduction

Glycans—a broad term describing carbohydrates, including oligosaccharides and polysaccharides—are the third class of important biological macromolecules following nucleic acids and proteins [1]. Glycans are found in all domains of life and in viruses. They can exist as free sugars, but are more commonly found as glycoconjugates, including proteoglycans, glycoproteins, and glycolipids. Glycans are involved in a wide variety of physiological functions and have implications in numerous infectious and non-infectious diseases, making them diagnostic and therapeutic targets [2]. Additionally, glycans are targeted in various biotechnological and industrial applications. The broad applications of glycans have spurred interest in the development of glycan binding proteins (GBPs).

GBPs include lectins, antibodies, pseudoenzymes, and carbohydrate-binding modules (CBMs). Lectins are non-immunoglobulin proteins containing at least one non-catalytic domain that exhibits reversible carbohydrate binding. CBMs are similar to lectins, but are small binding domains typically found in lectins or carbohydrate-active enzymes (CAZymes). CAZymes can be further classified into glycoside hydrolases, glycosyltransferases, polysaccharide lyases, and carbohydrate esterases—detailed information on these enzymes is available through the Carbohydrate Active Enzymes (CAZy) database [3]. Over time, some CAZymes have evolved into pseudoenzymes that have lost their catalytic activity but retain their glycan binding properties; these have been categorized separately from lectins as their overall structure is distinct. Antibodies against glycans are also found in nature, however glycans are generally poorly immunogenic, leading to low binding affinities and specificity of anti-glycan antibodies [4]. Despite this, some high-affinity anti-glycan antibodies have been developed [5,6]. The aforementioned protein categories have been used to create a variety of GBPs and their use in GBP engineering is discussed in Section 2.

One application for GBP engineering is towards the development of diagnostics and therapeutics. Glycan recognition is involved in a variety of bacterial and viral infections, which has led to the production of several diagnostic and therapeutic GBPs. For example, the glycan epitopes displayed on the envelope spike protein of the human immunodeficiency virus type-1 (HIV-1) are involved in immune system evasion [7]. More than a dozen lectins have been identified to exhibit anti-HIV properties and some are currently being further explored for therapeutic potential [8]. Similar to HIV-1, glycans are involved in immune system evasion in various other viral and microbial diseases—an excellent review has been published on the therapeutic value of GBPs in microbial infections [9].

GBPs also have the potential to target glycans that are involved in a variety of non-infectious diseases, such as diabetes [10], arthritis [11], and cancer—with glycans of the latter being the most well studied as biomarkers. Aberrant glycan profiles are a hallmark of malignant tumor transformations and are commonly targeted for cancer diagnostics and therapeutics [12]. The exact glycosylation patterns of tumors varies greatly, but include glycan epitopes that can be categorized as T-antigens, poly-*N*-acetyllactosamines (PLAs), Lewis antigens, and glycosaminoglycans, among others [13]. Cancer glycan epitopes have been recently reviewed elsewhere, and therefore will not be discussed in greater detail here [13].

GBP applications are not limited to the medical field and have seen broad applications in industrial settings and in biotechnologies. Lectins have been used as biological insecticides in genetically engineered crops and it has been suggested that this approach may reduce the use of synthetic insecticides—reducing risk to human health and the environment [14]. On another note, the CBM category of GBPs have applications in bioprocessing methods for affinity purification of biomolecules, using them in an immobilized selection step, or as affinity tags [15]. CBMs have also been used in textile industries to increase the efficiency of polysaccharide degrading enzymes—allowing for more efficient dyeing and printing on fabrics [16,17].

We have provided a summary of applications for engineered GBPs, but this list is by no means extensive—recent advances in glycomic techniques are expanding the known glycans that can be targeted for biotechnological, industrial, or medicinal purposes. In particular, development in laboratory techniques like lectin-arrays are leading to faster discovery of glycan biomarkers in human disease [18]. The rapidly growing literature on glycan targets has created a need for the development of novel diagnostic and therapeutic GBPs.

GBPs can be modified chemically or by using protein engineering techniques—here we will focus on the latter. The applications of GBPs range from diagnostics, therapeutics, and industrial settings to biotechnologies; however, the lack of known GBPs for specific glycans has created a need for GBPs with novel or altered binding specificities. Here we provide information that serves as a starting point for GBP engineering, with a focus on high-throughput directed evolution approaches. We address the topics of scaffold choice (Section 2), mutagenesis methods (Section 3), library screening and selection (Section 4), and characterization (Section 5), with a focus on how these methods have been used, and have yet to be used, for glycan binding protein engineering.

## 2. Glycan-Binding Protein Scaffolds

**Figure 1:**
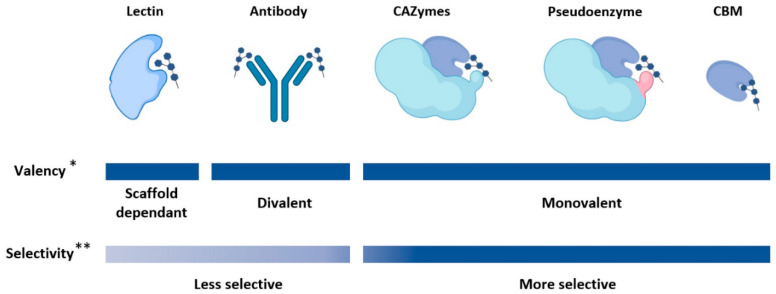
Valency and selectivity of protein scaffolds with glycan binding sites. The valency of lectins and antibody-based scaffold varies, as some lectins contain tandem repeat units and antibody-based scaffolds can be designed to contain only a single, or multiple variable fragments. Similarly, CBMs can be designed in tandem to increase valency. The selectivity of antibody-based scaffold is affected by the poor immunogenicity of carbohydrates. * It should be noted that the valency of lectins, antibodies, and CBMs can be altered with protein engineering. ** There are exceptions to this trend and the selectivity can be affected by valency.

Choosing the right protein scaffold is a crucial aspect of engineering a GBP with improved or novel binding properties. In this section we discuss examples of several protein scaffolds available for GBP engineering, including lectins, carbohydrate binding modules (CBMs), pseudoenzymes, carbohydrate-active enzymes (CAZymes), and antibody-based scaffolds (Figure 1). The definition of lectins has changed over the years [19,20], but can generally be defined as proteins that bind carbohydrates. Hence, most GBPs can be categorized as a lectin; however, for the purpose of this review, we have categorized certain GBPs separately from lectins due to their distinct characteristic folds and properties. A summary of the scaffolds discussed in this section, along with example scaffolds that have structural and binding data available, is available in Table 1.

### 2.1. Lectins

Lectins are carbohydrate binding proteins that are placed into sub-categories based on their folds and function: P-type, I-type, L-type, R-type, C-type, and galectins. Lectins display a wide variety of physiological functions and have biotechnological and biomedical applications—lectins have already been used in the detection and targeted treatments of human diseases such as cancer [21,22]. Here we provide a brief overview of lectins and some examples in GBP engineering. An excellent resource for detailed information on the various sub-categories of lectins can be found in the comprehensive text, *Essentials of Glycobiology* (specifically chapters 28 to 38) [1].

Generally, lectins have relatively low affinities for their glycan targets, with dissociation constants in the micromolar range [23,24]. This may be explained by the shallow binding interface that is observed in most lectins, causing more competitive solvent interactions. The shallow binding interface may also explain the promiscuous binding observed in lectins—glycans with similar structures often bind similar lectins. In nature, the low affinity problem is overcome by oligomerization and multivalency; in biological settings lectins tend to assemble into oligomeric structures containing multiple binding sites, allowing for higher affinities to be reached. The relatively low affinities and promiscuity of lectins in the monomeric state must be considered when selecting scaffolds for GBP engineering; however, lectins with improved binding specificity and affinity have been developed [25]. One advantage of using lectins over other protein scaffolds is that databases like UniLectin3D are available that can search for lectin scaffolds based on the glycan target [26].

### 2.2. Carbohydrate Binding Modules

Carbohydrate binding modules (CBMs) [27], also known as carbohydrate binding domains (CBDs), are non-catalytic protein domains generally found on carbohydrate-active enzymes (CAZymes). There is low sequence identity between CBMs [27], but there are conserved tertiary folds that are categorized based on their binding site topology as types A, B, or C [28]. The topologies of CBMs are characterized in type A by a planar hydrophobic surface, in type B by an extended binding cavity, and in type C by a short binding pocket—for more information on the structures of CBM types please see the extensive review by Armenta et al. [27]. For the purposes of GBP engineering, type A CBMs are suitable for binding insoluble, crystalline carbohydrates, due to the exposed planar binding interface [29]. In contrast, type B CBMs bind oligosaccharides [30], and type C CBMs bind mono and di-saccharides [31]. One attractive aspect of CBMs as GBP scaffolds is their modularity; due to their small size CBMs can be designed in tandem to increase specificity or allow for multiple binding targets. Additionally, there is a variety of well characterized CBMs that can be used as scaffolds—not surprisingly, CBMs have been used to engineer a variety of GBPs with altered binding characteristics [30,32,33,34].

### 2.3. Pseudoenzymes

In nature, a number of GBPs have evolved from enzymes through the loss of catalytic activity while retaining binding function. These can be defined as pseudoenzymes, which are catalytically inactive proteins related to ancestral enzymes [35]. Pseudoglycosidases are a type of pseudoenzyme that evolved from glycosidases (glycoside hydrolases). These proteins, which bind glycans but cannot hydrolyze glycosidic linkages, can also be characterized as lectins since glycan-binding is their primary function. A few notable examples of pseudoglycosidases that act as GBPs have been observed in nature. In animals, chitinase-like proteins such as the human YKL-39 are pseudoglycosidases (GH18 homologues) with enigmatic biological functions that have been shown to bind to chitooligosaccharides as part of their apparent role in modulating the innate immune response [36,37]. Another example in animals is found in α- and β-klotho proteins, which each make up part of a receptor complex responsive to fibroblast growth factors (FGFs), wherein catalytically inactive GH1-like tandem repeats of the klotho proteins bind to “sugar-mimicking motifs” of FGF19 and FGF21 [38]. In protozoans, the CyRPA protein of *Plasmodium falciparum*—part of the invasion complex that allows the malaria-causing parasite to bind and enter red blood cells—appears to be a catalytically inactive pseudoglycosidase related to GH33 sialidases [39,40,41]. Pseudoenzymes evolved from other types of enzymes can also bind to glycans. For example, PgaB in *E. coli* is a deacetylase that is involved in the formation of the partially deacetylated poly-1,6-*N*-acetylglucosamine component of the bacterium’s biofilm coat, and the protein consists of two tandem domains related to carbohydrate esterase family 4 (CE4), with the C-terminal domain being a catalytically inactive pseudoesterase involved in binding poly-1,6-*N*-acetylglucosamine [42].

Although pseudoenzymes can, in theory, be used as glycan binding scaffolds, there are no published works on engineering pseudoenzyme scaffolds into novel GBPs as of 2020. This may be due to a lack of known pseudoenzymes scaffolds but may also be due to the prevalence of mutagenesis techniques that allow for inactivation of enzymes. The use of enzymes as GBP scaffolds is discussed in greater detail in the following section.

### 2.4. Carbohydrate-Active Enzymes (CAZymes)

Carbohydrate-active enzymes (CAZymes) catalyze reactions that break down, assemble, or modify saccharides, and they are categorized based on their activities and further subdivided into families based on sequences. Categories include glycosyltransferase, glycoside hydrolase, polysaccharide lyase, carbohydrate esterase, and auxiliary activity families. The examples of pseudoenzymes from nature demonstrate that inactivation of CAZymes can result in proteins that bind to glycans but do not catalytically turn them over. Naturally, this suggests that inactivating CAZymes through artificial mutations may be an effective method to engineer novel GBPs. Generating a GBP from a CAZyme requires the inactivation of catalytic residues, which sometimes only requires the mutation of a single amino acid. This may make CAZymes an attractive scaffold for GBP engineering. An example of a nanomolar affinity GBP engineered by inactivation of a CAZyme can be seen in the site-specific mutation of a glycoside hydrolase from *E. coli* K1 bacteriophages. The GH58 endosialidase, Endo-NF, was mutated to generate a catalytically inactive GBP that still binds to polysialic acid with a dissociation constant (*K*_D_) of 191 nM [43,44]. This engineered GBP has been applied as a very sensitive tool for detecting polysialic acid [45,46]. In another example, mutation of a CE2 carbohydrate esterase from *Clostridium thermocellum* has also been shown to produce a catalytically inactive GBP with micromolar affinity [47]. A single amino acid replacement of the *Ct*CE2 enzyme not only abolished esterase activity, but increased the affinity to cellooligosaccharides nearly 8-fold, with the mutant binding to cellohexaose with a *K*_D_ of 4.1 μM.

The strategy of engineering GBPs by inactivating CAZymes has been developed and commercialized most notably by the biotech company Lectenz Bio, who have produced a variety of catalytically inactivated CAZymes, which they have dubbed “Lectenz^®^” (lectins engineered from enzymes) [48]. The company has produced several Lectenz^®^ through site-directed mutagenesis and computationally guided directed evolution. One advantage of using CAZyme scaffolds is that carbohydrate-processing enzymes tend to be more specific for their ligands than lectins, although this will vary between proteins.

### 2.5. Antibody-Based Scaffolds

Antibody-based scaffolds consist of immunoglobulin or immunoglobulin-like protein folds. A variety of antibody-based scaffolds are found in animals, but the most commonly used for developing antigen binding proteins are immunoglobulin G (IgG), and more recently, camelid antibodies [49]. The production of naturally occurring antibodies is time consuming and costly as it requires the immunization of an animal; however, antibody-based scaffolds have been engineered that circumvent the use of animals. These include, but are not limited to, antigen binding fragments (F_ab_) [50], single chain variable fragments (ScFvs) [51], diabodies [52], monobodies, and nanobodies [53]. There has been a concerted effort to produce antibodies against tumor-associated carbohydrate antigens (TACAs)—in total, antibodies have been designed for about 250 distinct glycan targets [54]. Antibody scaffolds offer certain advantages over lectins, including a larger binding interface for longer glycan epitopes, and generally more selective binding due to the complementary determining regions. However, glycans are poorly immunogenic and producing an anti-glycan antibody can be costly, labour intensive, and time consuming. Additionally, anti-glycan antibodies generally have lower affinities (*K*_D_ in the micromolar range) than protein-targeting antibodies (*K*_D_ in the nanomolar range). Within the last decade, phage display has provided methods for overcoming some of these limitations, resulting in antibodies with higher affinity for their glycan targets [55]. However, this approach still requires an initial scaffold obtained from immunization to be used as the base scaffold for improving affinity and selectivity.

### 2.6. Summary on Available GBP Scaffolds

Here we discussed the available protein scaffolds and some of their respective challenges and considerations when applied to GBP engineering. The scaffold that is chosen for GBP engineering will influence which mutagenesis techniques and selection methods are most appropriate. This brief overview provides a resource for glycobiologists who aim to design novel GBPs for specific glycan targets. Table 1 is by no means a complete scaffold list; it serves as a list of example scaffolds that are available and characteristics that need to be taken into consideration. Finding scaffolds ideal for a glycan of choice can be challenging and we recommend using UniLectin3D or equivalent GBP databases as starting point for finding potential scaffolds [26].

**Table 1:** Carbohydrate binding proteins (CBPs) with available structural and ligand binding information.

Scaffold Category	Scaffold Sub-Category	Description	Origin	Example Protein (PE)	PE Length	PE Ligand	PE Oligomeric State	PE Multivalency
Lectins	P-type	Lectin that binds to mannose 6-phosphate	Animal	Bovine CD-MPR binding domain [56]	154 aa	Mannose 6-Phosphate	Dimer	Monovalent
	I-type	Protein that is homologous to the immunoglobulin superfamily (IgSF)	Vertebrata	hCD22 domains 1-3 [57]	324 aa	Sialoglycans	Monomer	Monovalent
	L-type	Proteins that are structurally similar to lectins found in the seeds of leguminous plants	All domains of life and viruses	Concanavalin A [58,59]	237 aa	Trimannoside containing-oligosaccharides [59]	Oligomer	Divalent
	R-type	Proteins that are structurally similar to the carbohydrate recognition domain (CRD) in ricin	All domains of life and viruses	Ricin [60]	267 aa	β1,4 galactose, *N*-acetylgalactosamine	Dimer	Divalent
	C-type	Ca^2+^ dependant proteins that share a primary and secondary homology in their CRDs	Animal	C-type domain of murine DCIR2 [61]	129 aa	*N*-glycans	Monomer	Monovalent
	Galectin	Globular proteins that share primary structural homology in their CRDs	Animal	hGalectin-3 [62]	146 aa	*N*-acetyllactosamine	Monomer	Monovalent
Carbohydrate Binding Modules (CBMs)	Type A	Protein domain that binds to crystalline surfaces of cellulose and chitin	All domains of life and viruses	CBM from Cel7A [63]	36 aa	Cellulose	Monomer	Monovalent
	Type B	Protein domain that binds endo-glycan chains	All domains of life and viruses	CBM4-2 from xylanase [33]	150 aa	Xylans, β-glucans	Monomer	Monovalent
	Type C	Protein domain that binds exo-type glycan chains	All domains of life and viruses	Cp-CBM 32 of hexosaminidase [64]	150 aa	*N*-acetyllactosamine	Monomer	Monovalent
Pseudoenzymes	Pseudoglycosidase	Carbohydrate binding proteins that evolved from glycosidases but are no longer catalytically active	Possibly all domains of life*	hYKL-39 [36]	365 aa	Chitooligo-saccharides	Monomer	Monovalent
	Pseudoesterase	Carbohydrate binding proteins that evolved from carbohydrate esterases but are no longer catalytically active	Possibly all domains of life *	C-terminal domain of PgaB [42]	367 aa	Poly-1,6-*N*-acetylgluco-samine	Monomer	Monovalent
Carbohydrate- Active Enzymes (CAZymes)	Glycoside hydrolase	Enzymes that cleave glycosidic linkages	All domains of life and viruses	Endo-NF (GH58) [44]	811 aa	Polysialic acid	Trimer	Multivalent
	Carbohydrate esterase	Enzymes that hydrolyze ester linkages of acyl groups attached to carbohydrates	All domains of life and viruses	CtCE2 [47]	333 aa	Cellooligo-saccharides	Monomer	Monovalent
	Other CAZymes (glycosyltransferase, polysaccharide lyase, auxiliary activities)	Enzymes involved in the assembly, break-down, and modification of carbohydrates	All domains of life and viruses	−	−	−	−	−
Antibodies	N/A	Naturally or synthetically produced proteins with an immunoglobulin, or derived from an immunoglobulin-like structure	Vertebrata	hu3S193 [65]	LC: 219 aa HC: 222 aa	Lewis^Y^	Dimeric	Divalent

* Ancestral enzymes found in all domains of life.

## 3. Mutagenesis Methods for Library Generation

When a suitable protein scaffold is chosen it can be engineered into a novel glycan binding protein (GBP) using random, rational, or semi-rational mutagenesis. Random mutagenesis can generate large libraries for directed evolution approaches without structural information, whereas semi-rational and rational mutagenesis require structural data. There are various mutagenesis methods that fall into random, semi-rational, and rational mutagenesis, some of which can be used in combination. Here we provide a brief overview of these methods and examples of their uses for engineering GBPs.

### 3.1. Sequence Agnostic Random Mutagenesis

Random mutagenesis techniques that do not require any structural information and allow for mutation at any position within the protein-coding region of a gene can be considered “sequence agnostic”. Sequence (and structure) agnostic mutagenesis approaches are the methods of choice for library generation when no structural data is available. The mutant libraries generated in this way can be used for directed evolution, in combination with high-throughput screening or selection techniques (Section 4). Methods for random mutagenesis include error prone PCR (epPCR) [66], DNA shuffling [67], in vivo mutagenesis using mutator strains [68], and external mutagens [69] (Table 2).

A powerful and versatile yet straightforward technique, epPCR is the most common method for creating mutant libraries of a single gene. In epPCR, conditions are chosen to allow for a relatively high mutation rate by the DNA polymerase (i.e., low fidelity of replication). This can typically be achieved by adjusting the concentration of DNA polymerase and MgCl_2_, adding MnCl_2_, and adjusting the ratio of dNTPs, or by using an engineered DNA polymerase mutant with reduced fidelity [66]. As it is the most common mutagenesis technique, it comes as no surprise that epPCR has been applied to engineer novel GBPs. In one example from 2007, Yabe et al. cloned an earthworm galactose-binding lectin, EW29Ch, as the starting point for directed evolution wherein variants from successive generations were selected from mutant libraries generated by epPCR. This approach produced an engineered GBP specific for α2,6-sialic acid, a ligand not recognized by the parent protein [70].

In other examples, epPCR can also be combined with other mutagenesis techniques, such as DNA shuffling. DNA shuffling involves recombination of a population of homologous genes that have diverged either naturally or through laboratory mutagenesis of a parent (e.g., by epPCR). In this technique, random fragmentation of genes in a library (e.g., by DNase I digestion) is followed by PCR-based reassembly of overlapping fragments with sufficient homology, which effectively recombines mutations within the gene library [71]. Examples of DNA shuffling in combination with epPCR are seen in protein engineering efforts that have introduced mutations to the CBM of cyclodextrin glucanotransferase from *Bacillus* sp., and the glycan-binding regions of *N*-oligosaccharyltransferase from *Campylobacter jejuni* resulting in increased specificity and efficiency of those enzymes [72,73].

Alternative to the in vitro mutagenesis methods described above, one can perform in vivo mutagenesis on a target gene. These in vivo methods involve manipulating the DNA replication and repair machinery of the organism in which the target gene is cloned. For example, in mutator strains like *E. coli* XL1-red, which is deficient in three of the primary DNA repair pathways (carrying mutations *mutS*, *mutD*, and *mutT*), imperfect replication of DNA results in the accumulation of mutations in the cloned gene (along with the vector) [68]. In an example from 2011, Mendonça and Marana used in vivo mutagenesis in *E.coli* XL1-Red to alter the specificity of a β-glycosidase, *Sf*βGly, from *Spodoptera frugiperda* [74]. Mutants from a library generated in the mutator strain were screened for their specificity towards fucosides vs. glucosides, and several variants were identified that differed from the parent enzyme in their substrate preference. Given that glycosidases can serve as scaffolds for GBPs through their catalytic inactivation (Section 2.4), this can be a useful strategy for engineering novel GBPs. The advantage of mutator strains is that their use involves simple protocols, generally involving transformation of the mutator strain by a plasmid followed by propagation and plasmid recovery. However, mutator strains get progressively sick as they divide due to the deficiencies in their DNA repair mechanisms, and consequently this mutagenesis method often requires frequent re-transformations. Other in vivo mutagenesis methods use external mutagens such as UV radiation or mutagenic chemicals (e.g., ethyl methanesulfonate) which can avoid some of the challenges of maintaining mutator strains.

Regardless of the sequence agnostic random mutagenesis techniques used, one disadvantage is that the produced libraries only cover a small fraction of the possible mutations and require considerable effort to screen. Rational and semi-rational mutagenesis can be more efficient at producing effective mutations based on the structural and functional information when it is available.

### 3.2. Rational and Semi-Rational Mutagenesis

Rational and semi-rational mutagenesis can produce smaller libraries than sequence agnostic random mutagenesis techniques, while simultaneously focusing on mutations that are more likely to impact protein function in a desirable way. Rational mutagenesis, as defined herein, involves making precise amino acid substitutions to a protein scaffold based on its structural data, whereas semi-rational mutagenesis uses the structural data to target specific sites that are then randomized. Some degree of rationality is always used to limit the sequence space that can be covered in mutagenesis, making it difficult to clearly distinguish between semi-rational and rational design—therefore, we have grouped these techniques together. Both techniques make use of various site-directed mutagenesis (SDM) methods (Table 3).

SDM methods are applied to engineering binding proteins when detailed structural information is known about the binding site, or binding-determining regions of the scaffold protein. The most common and efficient way to target specific sites is by PCR—desired single point mutations are included in the primers that amplify the gene of interest. PCR based SDM is one of the most common SDM methods applied in protein engineering and variations of PCR site-directed mutagenesis have been successfully applied in engineering GBPs in several examples including altering the binding specificity of a fucose-binding lectin PA-IIL [75] and increasing the affinity of a tail spike protein Sf6 to the glycans comprising bacterial O-antigens [76]. SDM has also been used to inactivate a streptococcal endo-*N*-acetylglucosaminidase (EndoS) [77] and an *E. coli* K1 bacteriophage endosialidase (EndoNF) to dissociate binding from hydrolytic activity [43,44] and produce novel GBPs for specific glycan detection [45,46].

One drawback of standard SDM approaches is that they only sample a few defined mutations that may or may not result in the desired protein function; hence a sub-category of SDM, site-saturation mutagenesis (SSM) is often used to increase the sample space. Instead of a straightforward replacement of one amino acid for another, SSM randomizes a specific codon, or short sequence of codons, to produce libraries of mutants with all possible amino acid substitutions (or a subset of possible substitutions) at the targeted positions [78]. Screening of such libraries allows for the identification of ideal amino acid replacements for those positions [79]. Although there are various SSM techniques, they all rely on site-directed mutagenesis PCR using degenerate codons. SSM has been successfully used to alter the glycan-binding specificity of a galectin towards α(2,3)-linked sialic acid in a single mutagenesis step [80]. This demonstrates the efficiency of SSM when compared to sequence agnostic random mutagenesis techniques that often require multiple cycles of mutagenesis to produce a desired mutant.

While directed mutagenesis can result in site-specific replacement of codons for targeted amino acid positions, it can also be employed to replace longer sequences of DNA for motif- or domain-swapping mutagenesis (substituting long strings of amino acids). Replacement DNA cassettes can be introduced by restriction digestion and ligation, or by overlap extension PCR to substitute a parental DNA fragment. In one example, motif-swapping has been used to alter the binding specificity of galactose-specific *Bauhinia purpurea* lectin by switching nine amino acids in the binding region with the corresponding nine residues present in the mannose-specific *Lens culinaris* lectin, generating a chimeric lectin that has a unique carbohydrate-binding specificity not observed in the parental proteins [81].

**Table 3:** Overview of site-directed mutagenesis techniques.

Method	Definition	Pros	Cons	References
PCR site-directed mutagenesis	Primers containing the desired mutation(s) are used to alter the original gene	Not limited by the availability of nearby restriction enzyme cut sites	Primer design can be complicated when introducing multiple mutations	[43,44,45,46,75,76,77,82]
Site-saturation mutagenesis	A set of codons is substituted with every amino acid using degenerate codons	Allows for the screening of ideal amino acids at different positions	Mutation bias from the degenerate codon	[78,80]
Motif- and domain-swapping	Uses a “cassette” DNA fragment containing the mutations, which replaces the unmutated segment in the original gene	High mutation efficiency	Is limited by the domains/motifs that are used.	[81,83]

### 3.3. Computational Tools for Rational and Semi-Rational Mutagenesis

In rational and semi-rational approaches, application of the aforementioned mutagenesis methods is guided by various computational approaches to identify positions for directed mutagenesis and predict beneficial mutations. These include homology modeling, molecular dynamics, deep learning, and tools for designing focused libraries (Table 4). Here we provide a brief overview of these computational methods and their applications in GBP engineering.

The 3D structure of a protein provides valuable information to guide its engineering, allowing one to identify residues that interact (directly or indirectly) with a ligand and to focus mutations at positions where they may be most effective. For proteins without solved structures, a homology model may be used where possible as an imperfect substitute. Homology modelling is one of the most common computational methods used for protein engineering, as it allows for the construction of an atomic resolution model for a target protein of unknown structure, provided that the structure of a sequence-homologous protein has been solved. The model is based on the structural data of homologous proteins and is generally considered reliable if there is more than 50% sequence identity between the target and homologue. In certain cases, homology models allow for precise editing of a protein. Using a homology model, Lienenmann et al. were able to identify and inactivate the catalytic residue in the *Trichoderma harzianum* chitinase, Chit42—turning the enzyme into a GBP [84]. However, X-ray crystal structures and homology models only provide a snapshot of the protein—unlike molecular dynamics (MD) simulations.

Molecular dynamics simulations generate a set of possible conformations based on the protein structure. One advantage of MD simulations is that they can identify flexible regions that may alter the protein activity. There are plenty of MD program packages available to use, with some of the most common being YASSA, MOE, Enlighten2, and GROMACS. In recent work on GBP engineering, Kunstmann et al. used MD simulations generated with the GROMACS 4.5.5 package to accurately link mutations in the tail-spike protein of bacteriophage Sf6 to varying affinities towards glycans of the O-antigens on *Shigella flexneri* (which is host to the phage) [76]. Yet being able to make such predictions accurately is rare and it is more common to identify beneficial mutations through screening focused libraries.

The insights gathered from analysis of protein structures, homology models, and MD simulations can be used in designing focused libraries of mutants. Focused libraries—in which specific positions are targeted for mutation (e.g., by SSM)—can be created with the assistance of computer tools for identifying amino acid sequences that are more likely to be involved in stability, catalytic activity, or specificity. These enriched libraries improve the efficiency of directed evolution by reducing the library size and have been used to design proteins with increased selectivity [85,86] and specificity [87]. Multiple tools are available for focused library design, such as CASTER and HotSpot Wizard 2.0, with the latter being a more accessible web-based tool that requires less bioinformatical knowledge.

One method of creating focused libraries that has been advancing rapidly is deep learning. Deep learning algorithms are a form of machine learning that trains artificial neural networks to recognize patterns in complex datasets such as sequencing data to predict properties of novel or uncharacterized proteins. Although deep learning is a fairly new technique, it has already been applied to lectin engineering. In 2012 Stephen et al. used a deep learning algorithm called CPred to produce a starch-binding domain with altered selectivity by predicting the effects of circular permutation [88,89].

Here we have only briefly covered some computational methods that are available for protein engineering of GBP—for more details on computational approaches to protein design, Kuhlman and Bradley provide an excellent review [90].

**Table 4:** Example of computational approaches used in protein engineering.

Computational Approach	Definition	Examples	Utility in Protein Engineering	References
Homology model	Constructs an atomic resolution model of a protein based on available structural data of related homologous proteins.	Phyre2, ROBETTA, SWISS-MODEL	Predicted structures can be used in other computational methods, such as docking and molecular dynamics simulations.	[84]
Molecular Dynamics	Predicts the conformational energy landscape available to a protein based on the structure.	YASARA, Enlighten2, MOE, GROMACS	Dynamics can indicate how certain mutations can affect the behavior of protein such as folding, stability, ligand interaction and enzymatic activity.	[76,91,92]
Deep learning	Uses known protein sequences and properties to predict the properties of uncharacterized or novel proteins.	CPred, UniRep	Allows for the generation of more efficiency mutant libraries, by focusing on the most promising candidates.	[88,89,93]

## 4. Library Selection and Screening Methods for GBPs

Library display techniques such as phage, yeast, ribosome, and mRNA display allow for the high-throughput selection of specific binding proteins from libraries of mutant variants. Regardless of the method used, there are three general steps involved: (1) library generation, (2) biopanning, and (3) characterization of variants. This section will focus on biopanning, since the library generation was previously described (Section 3), and characterization is dependent on the target protein (Section 5).

### 4.1. Phage Display

**Figure 2:**
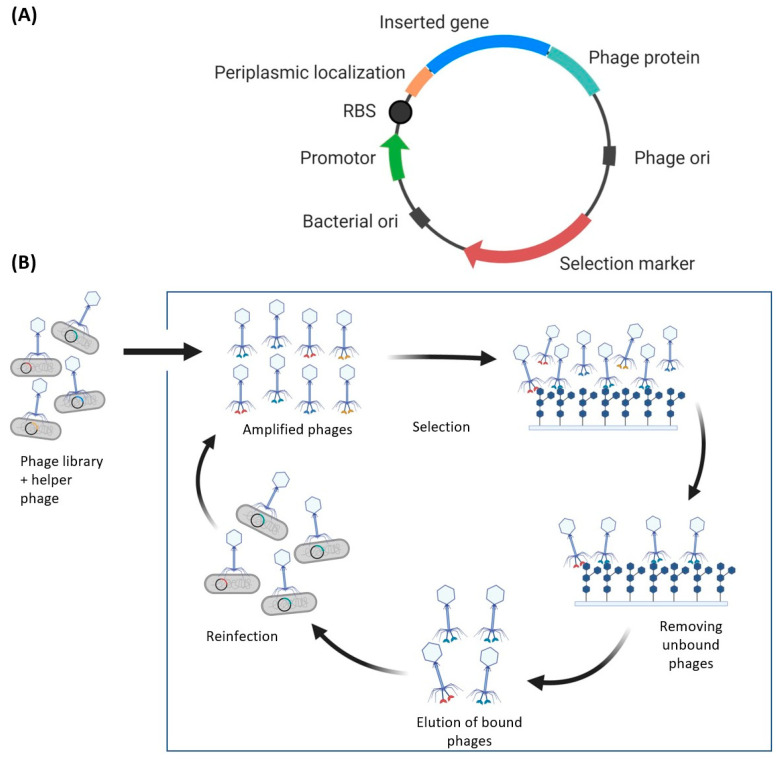
(**A**) Example phagemid design for phage display. The phage plasmid requires two origins of replications, one for *E. coli* (the host) and one for the phage. Additionally, attaching a periplasmic localization signal to the target gene may improve the display, although this may depend on the appended phage protein. The phage protein is drawn attached to the C-terminus; however, it may be required to append it by the N-terminus—linkage is dependent on the phage protein. (**B**) Simplified schematic of phage display. The protein library is transformed into *E. coli* and amplified using helper phages, resulting in phages displaying the protein library. The phages can then be tested for antigen binding, using surfaces or beads that contain the antigen. A washing step removes unbound phages, and positive binders can be eluted and used for reinfection and amplification of the selected phages. This bio-panning cycle is repeated for multiple rounds and remaining phages are characterized and recombinantly expressed.

Phage display is a high throughput selection technique for large libraries of mutant proteins that links the protein library to the coat proteins of bacteriophages (most commonly M13 filamentous phage, T4, T7, and λ phage). This method displays protein libraries on the surface of bacteriophages by encoding the library into a phage-derived circular DNA vector—a phagemid. The phagemid encodes a coat protein fused to a mutant protein of interest, which links the phenotype of the protein (binding ability, enzymatic activity etc.) to its genotype. Typical elements of phagemids include bacterial and phage origins of replication, a selection marker, and the gene of the displayed protein appended to a phage protein-coding gene, which may also encode a periplasmic localization signal depending on the phage protein (Figure 2A). Displayed proteins can be selected for desired function then genotyped using high-throughput selection methods followed by sequencing. This section provides a brief overview of phage display; an extensive review is published in Chapter 3, volume 580 of *Methods of Enzymology* [94].

A phagemid library encoding a pool of mutants produced by one or more mutagenesis methods (Section 3) is used to transfect *E. coli*—the transformation efficiency limits the protein library diversity between 10^7^ to 10^9^ unique protein sequences [95]. Typically, the phagemid does not encode viral proteins that are needed for viral propagation. Hence, a helper phage that expresses viral proteins essential for efficient viral propagation is used to ensure high copy numbers of the phage library. Methods have been developed that eliminate the need for helper phages, but they are not as commonly used [96]. Regardless of whether helper phages are used, the amplification of the phage library is the beginning of the biopanning cycle that involves binding, washing, elution, reinfection, and phage amplification steps (Figure 2B). The phage library can then be used to test for binding to the target of interest and successful binders may be used to repeat the cycle using phage reinfection and amplification.

With regards to glycan binding proteins, phage display has been used to produce a single chain variable fragment (ScFv) against T-antigens—a glycan marker of many adenocarcinomas [97]. The anti T-antigen ScFv was selected from a library of human ScFvs and successfully bound T-antigens with micromolar affinities. Likewise CBM4-2 of *Rhodothermus marinus* xylanase Xyn10A (18 kDa) has been used as a scaffold in phage display to engineer new binding properties towards xylan, and the glycoprotein IgG4 [33]. An advantage of phage display over the other display methods is that it has been well optimized as it is the oldest and most common display technique. However, phage display does not allow for simple incorporation of many post-translational modifications (e.g., protein glycosylation) without chemical manipulation of the phage after amplification [98]—in contrast to yeast display.

### 4.2. Yeast Display

**Figure 3:**
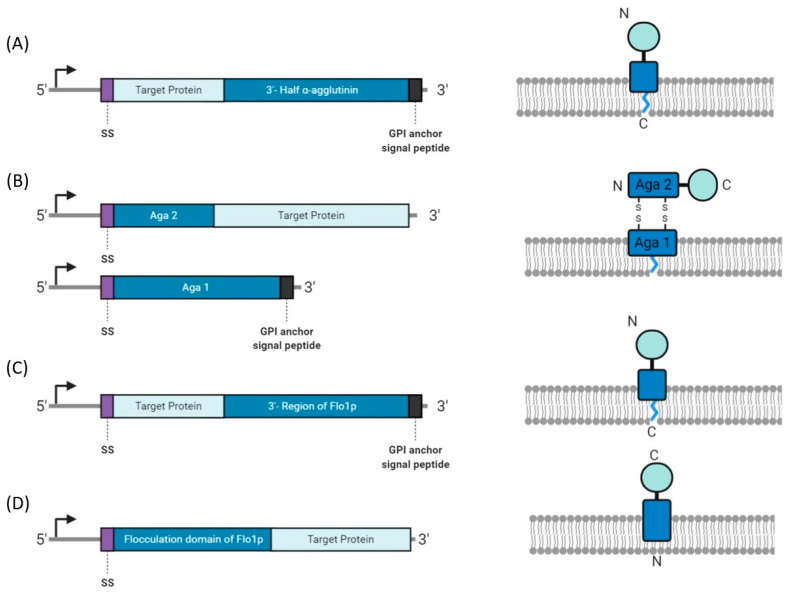
Construct designs for yeast display systems. (**A**) α-agglutinin is fused to the C-terminus of the target protein and anchored to the cell membrane by a glycosylphosphatidylinositol (GPI) anchor. (**B**) a-agglutinin (Aga) is a dimer, with Aga2 linked to the N-terminus of the target protein. Aga1 is membrane bound by a GPI anchor and forms disulfide bonds with Aga2. (**C**) The C-terminal domain of Flo1p is linked to the C-terminal region of the target protein. (**D**) The N-terminal domain of the Flo1p is linked to the N-terminal region of the target protein.

In yeast display, the proteins of interest are fused to cell surface proteins, most commonly α-agglutinin (Agα1), a-agglutinin (Aga1p-Aga2p), or flocculin (Figure 3). Depending on the anchor protein, the target protein can be fused to the N- or C-terminus, resulting in display of up to 100,000 copies [99]. The target protein is generally tethered to the anchor protein at the terminus farthest from the functional region, or binding region of the protein, to avoid interference. Additionally, the target protein can be flanked by protein tags for simplified purification and detection methods.

Yeast display is commonly used for antibody-like proteins, such as ScFvs, Fabs, and monobodies. In 2013, Hong et al. used yeast display to produce monoclonal lamprey antibodies (lambodies) against several tumor associated carbohydrate antigens, Lewis antigens, and several glycoproteins [100]. More recently, a fully human ScFv was produced that could bind an epithelial tumor associated *N*-glycoform of periostin, further demonstrating the value of yeast display in engineering GBPs [101]. One advantage of yeast display over phage and in vitro display methods is the ability to include native (or similar to native) post-translational modifications, which can impact the protein’s fold and function. Additionally, yeast display can take advantage of fluorescence-activated cell sorting (FACS) for efficient library screening (after the cells bind to a fluorescently tagged target ligand). However, yeast display is more time intensive than in vitro display methods, and the mutant library size is restricted to 10^7^ unique mutants—the smallest out of all display methods discussed in this review.

### 4.3. Ribosome Display

**Figure 4:**
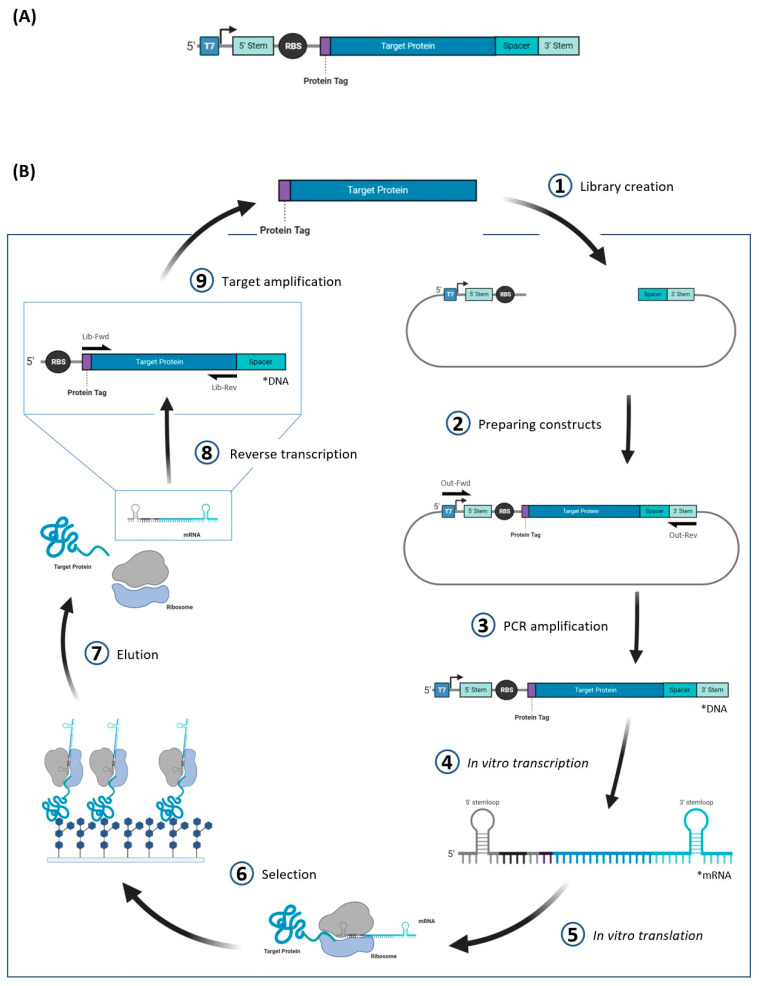
Overview of the ribosome display method. (**A**) Construct design for ribosome display. The sequence that is transcribed (4) during the ribosome display contains 5′ and 3′ stem loop regions that help prevent degradation of the subsequent mRNA. (**B**) Cyclic representation of ribosome display. The plasmid used for ribosome display typically includes the promoter, stem loop regions, RBS, and spacer region.

Unlike yeast display and phage display, ribosome display is performed in a cell-free process that involves in vitro transcription and translation, and it is not limited by transformation efficiencies. In ribosome display the target protein is linked to its mRNA in a ribosome-mRNA-target protein complex. This complex is established during in vitro translation, as the mRNA of the target protein lacks a stop codon—preventing the dissociation of the mRNA and protein from the ribosome. The design of the DNA construct, including the gene coding for the target protein, requires an upstream ribosomal binding site (RBS) and a downstream spacer (Figure 4A). The DNA construct is transcribed in vitro to mRNA, and the spacer sequence on the mRNA allows the protein of interest to sit outside of the ribosome tunnel, and fold properly, while remaining bound to the ribosome after it is translated in vitro. It is recommended that the construct include regions that will be transcribed into stemloops upstream of the RBS and downstream of the spacer—the secondary structure of the mRNA stemloops can prevent mRNA degradation and increase ribosome efficiency up to 15-fold [102]. Figure 4B provides an overview of the entire mRNA display cycle.

In one example of ribosome display applied to GBP engineering, a sialic acid-binding lectin for use in analytical microarrays has been engineered from a galactose-binding lectin using ribosome display in combination with epPCR [70]. One advantage of cell-free display methods like ribosome display is the ease with which PCR-based mutagenesis methods can be implemented after each selection round—allowing for multiple generations of mutants to be evolved through iterative cycles of randomization and selection, mimicking the natural evolutionary process. However, during ribosome display the protein of interest is bound to an enormous 2.7 MDa ribosome complex, which may interfere with the target protein’s characteristics. A cell-free display technique that does not link the target protein to a macromolecule and may have less interference is mRNA display.

### 4.4. mRNA Display

**Figure 5:**
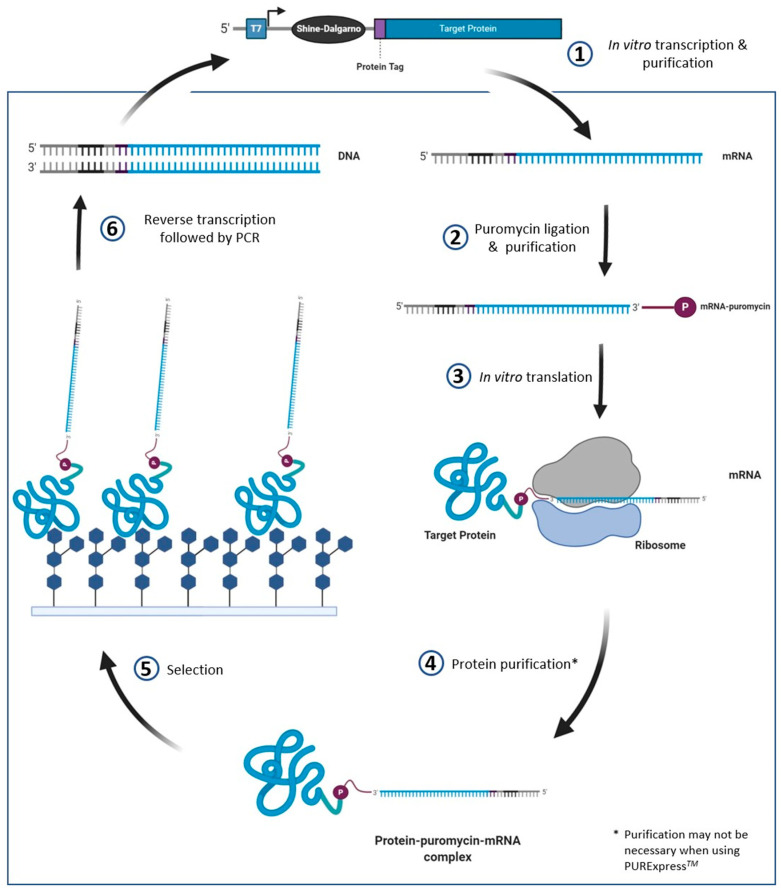
Overview of the mRNA display method. The initial construct design has to contain a promoter (e.g., T7) and RBS (e.g., Shine Dalgarno) specific to the cell free expression system used. The puromycin mimics an aminoacyl tRNA which causes the mRNA to be linked to the nascent protein during in vitro translation. Once translated, the mRNA-protein complex is screened for glycan binding. The mRNA attached to the bound proteins is reverse transcribed and PCR amplified—allowing for simplified sequence detection.

mRNA display is a cell-free display method in which the protein of interest is covalently linked to the 3′ end of its own mRNA. DNA constructs for mRNA display require a promoter (commonly the T7 promoter for an *E. coli*-derived system, but may vary depending on the cell-free expression system) to recruit RNA polymerase for in vitro transcription, and a ribosomal binding site in the 5′ UTR, allowing for in vitro translation of the protein. The mRNA is transcribed in vitro, then enzymatically or photochemically ligated to a DNA-puromycin linker [103,104]. During in vitro translation, the puromycin mimics an aminoacyl-tRNA and forms a bond with the nascent peptide. The resulting protein-mRNA complex can then be used in selection methods to identify binding interactions—an overview of the mRNA display process is provided in Figure 5.

The mRNA-protein linkage allows for simplified sequence detection following selection—the mRNA is reverse transcribed allowing for double-stranded cDNA to then be amplified by PCR. The resulting DNA can then be sent for next generation sequencing (NGS)—the sequencing data can be used to determine which proteins are enriched. As previously mentioned, one advantage of mRNA display over ribosome display is that it does not require the ribosome complex to be bound to the protein; hence interference is less likely. A review on mRNA display has recently been published, and discusses the topic in much greater detail than we will cover here [105]. However, we do note that as of 2020, no reports of mRNA display applied to the engineering novel GBPs have been published. It should also be noted that cell-free expression systems, like mRNA display and ribosome display, do not typically include protein folding chaperones, which may decrease the yield of properly folded proteins. Chaperones such as the *E. coli* proteins DnaK and GroEL can be added to cell free expression systems to increase the yield of functional proteins; however, they may not act as chaperones for every protein product and optimization would be required [106].

### 4.5. Glycan Immobilization Strategies for GBP Selection

Common to the range of selection methods to identify binding proteins, which involve different display techniques, is the need for an immobilized target. Various glycan immobilization strategies may be used for the selection and purification of GBPs. Glycan targets can be bound to a variety of solid supports including agarose resin [70], polymer-coated superparamagnetic particles (e.g., Dynabeads) [107], or the wells of standard microtiter plates [45,108]. This can be done using a number of chemical approaches such as amine coupling and click-chemistry.

Glycoproteins may serve as a convenient source of target glycans which can be fixed to a solid support. The entire protein along with its glycan modifications may be immobilized by covalent attachment to a chromatographic resin such as agarose. Glycoproteins such as bovine fetuin are readily available materials for cell biology and can be linked by amine coupling to resins that have been functionalized with, for example, aldehyde groups, cyanate esters, or *N*-hydroxysuccinimide esters. Fetuin-agarose produced in this fashion has been applied in the selection cycle for the directed evolution of a novel sialic acid-binding protein in a process involving ribosome display (Section 4.3) [70].

Coated magnetic particles, such as Dynabeads, can serve as another sort of solid support for target glycans. These versatile beads are used (often with the aid of automated magnetic particle handling robots) in the pull-down of proteins and nucleic acids. The use of glycan-coated Dynabeads has been demonstrated for specific pull-down of GBPs. Hence, Dynabead-linked carbohydrates can be used in a selection process for engineered GBPs. In a study by Liebroth et al. [107], Lewis^X^ and *N*-acetyllactosamine were immobilized to Dynabeads through a bovine serum albumin carrier, and these coated beads were then used to test the binding of TAG-1, Contactin, and NCAM120 (three lectin-like neuronal receptors). In pull-down assays carried out on a mixture of cellular proteins, it was determined that TAG-1 makes specific interactions with Lewis^X^ (but not *N*-acetyllactosamine), while the other two proteins do not. In a similar fashion, glycan-functionalized Dynabeads could be used to pull down binders to a target glycan from a library of GBPs, removing non-binders as part of a selection process.

A useful approach to link target glycans to solid support resins or surfaces involves “click” chemistry. Click chemistry describes a set of water-compatible, biocompatible reactions that can link two appropriately tagged reagents together in high yield (e.g., using azide-alkyne cycloaddition). Here we will focus on azide-alkyne based click chemistry, since it is a widely adapted tool in glycobiology research, seeing broad uses in applications including in vivo glycoengineering [109,110] and glycan labelling [108]. Notably, click chemistry has been used to immobilize functionalized glycans onto the surface of wells in microtitre plates. This has enabled high-throughput plate-based assays for glycosyltransferases exhibiting, for example, fucosyltransferase [111] or polysialyltransferase activity [45] on immobilized oligosaccaccharide substrates, detected through specific binding of the enzyme-product by tagged GBPs. Solid supports coated with immobilized glycans such as these could also be used for selection of GBPs.

## 5. Binding Characterization of GBPs

The most common binding characterization tools used for engineered GBPs are surface plasmon resonance (SPR) and titration calorimetry. SPR and titration calorimetry are both non-destructive method for determining binding characteristics; however, SPR requires the binding partner to be immobilized on a surface whereas titration calorimetry is done in solution. Specific applications of affinity chromatography using immobilized ligands have also proven useful in measuring GBP binding affinity, although this method is less accurate.

### 5.1. Frontal Affinity Chromatography

Using chromatographic resins functionalized with immobilized ligands, frontal affinity chromatography is an analytical technique that can be used to measure the binding interactions between molecular species. Application of this technique can be used to determine the binding constants of binding proteins of a wide range of equilibrium constants [112]. In particular, it has proven a useful tool in measuring protein-glycan interactions [113]. As a solution containing a GBP flows through a column packed with a glycan-functionalized resin, the degree to which the GBP is slowed by its interaction with the immobilized glycan can be used to measure binding interactions. Custom affinity resins with specific glycans can be generated, for example, as described in this review (Section 4.5). This approach has been demonstrated by Yabe et al. to characterize the binding of an engineered lectin to the sialic acid ligand to which it was tailored [70]. One advantage of affinity chromatography is that it can also be used to select and purify a protein target, yet the binding kinetics determined are not as accurate as SPR or titration calorimetry.

### 5.2. Surface Plasmon Resonance

**Figure 6:**
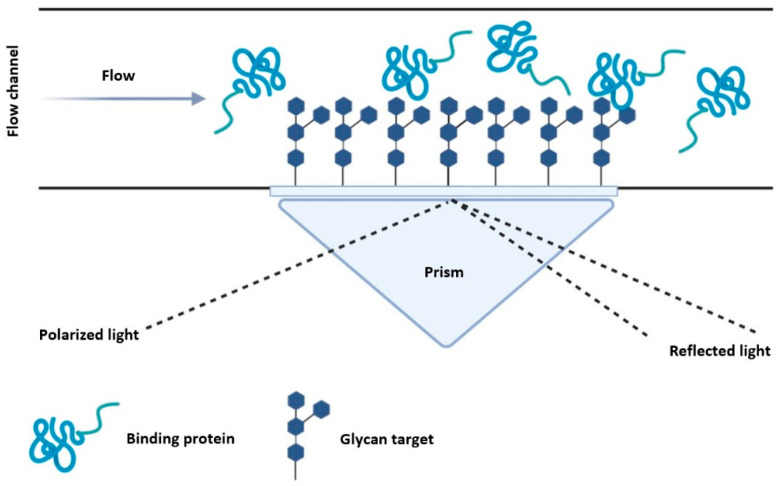
Overview of SPR with glycan labelled surfaces. Glycans are bound to a metallic surface inside a flow chamber and a GBP solution is introduced allowing the protein to bind. A light source excites the metallic surface, and the angle of the reflected light is relative to the mass at the surface, which enables binding kinetics to be determined.

Surface plasmon resonance (SPR) techniques measure the frequency of the electromagnetic oscillations on a metal surface, by exciting the surface electrons using a light source. The angle of the reflected light is influenced by the mass at the surface, hence mass changes on the surface can be measured based on the change in the reflected angle [114]. Glycan-labelled metal surfaces can therefore be measured for protein binding based on the change of the reflected angle (Figure 6). Moreover, SPR can measure binding interactions in real time—allowing for the determination of association and dissociation rate constants.

SPR has already been applied in glycobiology research to screen GBPs for glycan binding [115], produce a mannose biosensor capable of detecting nM concentrations of lectins [116], and for comparing glycan binding of lectin mutants [84]. The main advantage of SPR over other analytical techniques is the ability to provide real-time kinetic data for glycan-protein interactions, without the need for labelling methods. However, the equipment and specialized knowledge needed to apply SPR methods can prohibit the use of these techniques. One of the main challenges is attaching the glycans or GBPs to the surface. For glycans, protocols have been developed to attach phenoxy-derived sugars [117]. Immobilization of GBPs to the surface can be done in various approaches, including amine coupling, nickel affinity for His-tagged proteins, and streptavidin-binding for biotinylated proteins. Here we do not cover the various SPR techniques in detail—for a more detailed review on SPR and available surface labelling methods please refer to the Handbook of Surface Plasmon Resonance [114].

### 5.3. Titration Calorimetry

Isothermal titration calorimetry (ITC) can be used to screen GBPs for glycan binding by measuring changes in heat that occur during binding. Based on the heat changes, the enthalpy, entropy, stoichiometry, and binding constants can be determined. ITC has been used to screen the altered binding affinities of a mutated fucose lectin PA-IIL—providing evidence for the location of the specificity binding loop of the protein [75]. Additionally, ITC was used to determine the binding affinities of a mutated starch binding domain (CP90) for longer carbon chain starches [89]. Overall, this technique provides a non-destructive way to determine binding affinities; however, ITC is not suitable with high-throughput approaches since each mutant requires a separate ITC chamber.

## 6. Current Limitations in Lectin Engineering

Despite the available techniques for GBPs engineering, there are several factors that bottleneck the process, including the availability of glycans, the glycan binding interactions with the scaffolds, and the lack of available glycosylation profiles for cells and proteins.

### 6.1. Glycan Availability

One limiting factor is the availability of glycans—screening and selection methods to engineer a GBP for a specific glycan require the glycan to be available in high purity and quantity. Due to the large variety and complexity of glycans, only some glycan epitopes are available for purchase through vendors. Unlike oligopeptides and oligonucleotides synthesis, there are no general protocols for the synthesis of complex glycans—the development of chemical synthesis protocol for a single glycan can be time consuming and expensive. However, recent advances in enzymatic and chemoenzymatic synthesis of glycans using recombinant enzymes are providing a path for affordable and efficient production of glycans. Chemoenzymatic synthesis of glycans requires synthetic precursors, a series of glycosyltransferases (GTs), and protection and de-protection steps [118]—this approach has been shown to be more affordable than purely chemical approaches [119]. Additionally, the expanding database of characterized glycosyltransferases opens the possibilities for more glycan structures to be produced enzymatically. Enzymatic synthesis may expand on the available glycan targets available for GBP engineering, yet another bottleneck is in the interactions that a GBP scaffold needs to make with a glycan target.

### 6.2. Glycan Binding Protein Scaffolds

Glycans contain subtle stereochemical and regiochemical differences between isomers that would need to be distinguished by the binding protein. Additionally, glycans form extensive hydrogen bonding networks in water that would have to be broken by the GBP, making the interactions less favorable. In nature, lectins are used to bind complex carbohydrate structures with high specificity, however lectins with higher avidity for their targets are generally multivalent. The multivalency allows for stronger binding with its target but results in larger protein complexes that are more difficult to produce and hence less ideal for biotechnology applications. Unlike multivalent lectins, peptide aptamers and cyclic peptides are smaller and easier to produce, but do not provide large enough interfaces for complex glycan interactions, making them less ideal for glycan targets. Hence, there is a need in GBP engineering for protein scaffolds with large binding interfaces that are adaptable for biotechnological applications.

As high-throughput protein engineering methods become more common, future efforts may provide protein scaffolds for GBP engineering that bind glycans with higher affinity and avidity than what is currently available. Recently, designed ankyrin repeat proteins (DARPins) have been produced as highly stable, small molecular weight, modular scaffolds [120]. DARPins are based on ankyrin proteins, some of which have glycan binding pockets [121]; however, as of 2020, DARPins have yet to be used for GBP engineering. Due to their modular design that allows for multiple binding interfaces, these proteins may be ideal for glycan targets.

## 7. Conclusions

Engineered GBPs have broad applications in a wide variety of fields, including diagnostic, therapeutics, and biotechnology. GBPs that have been engineered so far have already been used in all the previously mentioned fields and as the field of glycobiology advances we expect more applications to become apparent. Although current methods are available to engineering GBPs, we are still limited by the number of available glycan structures that can be produced in pure quantities, and in the specificity and selectivity of the scaffolds. Additional research is needed to focus on glycan synthesis and on engineering novel scaffolds for glycan binding that provide larger binding interfaces to increase the specificity and selectivity of GBPs.

## Figures and Tables

**Table 2:** Overview of random mutagenesis methods.

Method	Definition	Pros	Cons	References
Error prone PCR (epPCR)	epPCR relies on increased error rate of the polymerase	Efficient amplification of mutants	Library size is limited by cloning efficiency	[66]
DNA shuffling	DNA shuffling randomly recombines point mutations during PCR	Can be followed up with epPCR	Mutation efficiency is highly dependant on the shuffled library	[67]
In vivo mutagenesis	Mutations are introduced in bacteria using chemical or physical means, chemical mutagens or mutators strains	Wider variety of mutations without bias	More labor intensive, mutator strains get progressively sick from mutations	[69]
